# Supplementary material for: Improving the Efficacy and Accessibility of Intracranial Viral Vector Delivery in Non-Human Primates
Source: Pharmaceutics. 2022 Jul 8;14(7):1435. doi: 10.3390/pharmaceutics14071435 (PMC9323200; doi:10.3390/pharmaceutics14071435)
Supplement: Supplementary file 1 [file pharmaceutics-14-01435-s001.zip › pharmaceutics-1783359-supplementary.pdf]

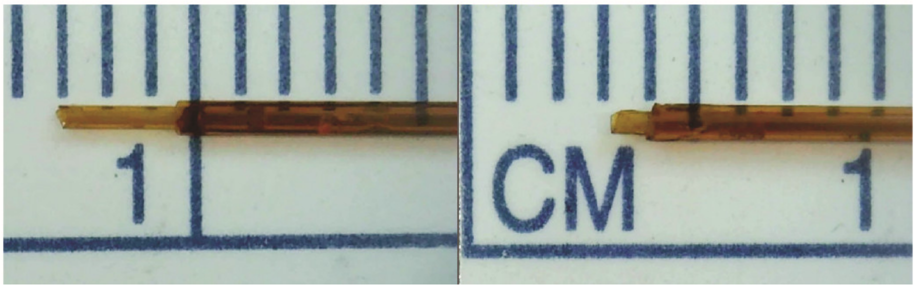

Figure S1. 3-mm (left) and 1-mm (right) stepped-tip cannulas

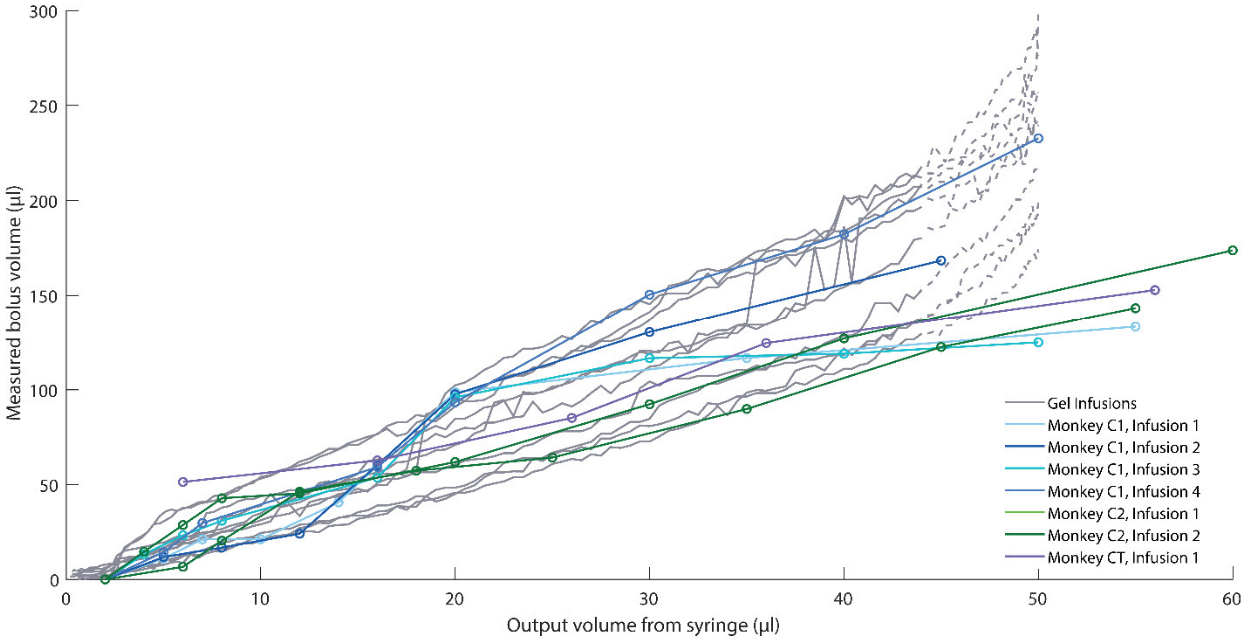

Figure S2. Comparison of agar and MRI cortical CED. This figure is the same as Figure 4B with the addition of data (dashed lines) from 44 µl to 50 µl which were omitted from statistical analysis due to their progressively steep upward trend which did not align with the MRI data. MRI data has been previously published [8].

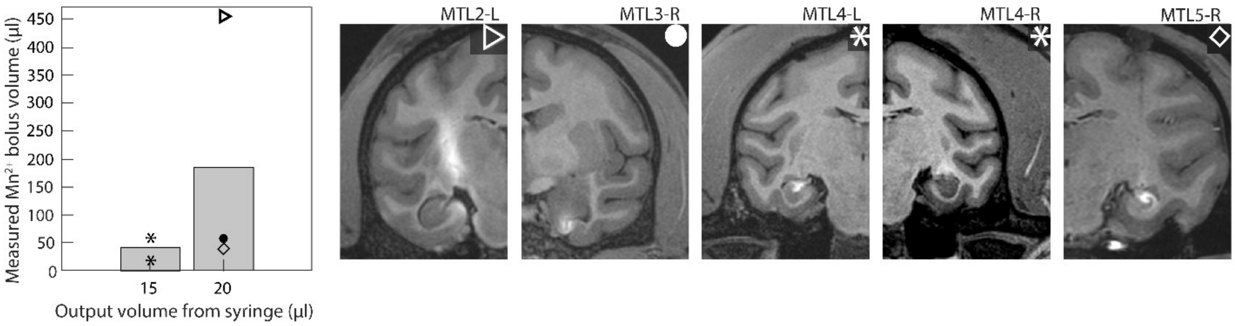

Figure S3. In addition to the deep infusions presented in Figure 6A, some infusions were not visually similar to cortical and thalamic data. These deep CED infusions were also made into the hippocampus, entorhinal cortex, and the tail of the caudate nucleus. Bar plots show the mean value of measured Mn^{2+} bolus seen in next-day, post-operative MRIs, with individual data points overlaid. Corresponding MRI slices in the coronal plane are shown. Shape labels correspond to each subject contributing to the data.
